# Estimating *Neospora caninum* prevalence in wildlife populations using Bayesian inference

**DOI:** 10.1002/ece3.2050

**Published:** 2016-03-02

**Authors:** Karla Moreno‐Torres, Barbara Wolfe, William Saville, Rebecca Garabed

**Affiliations:** ^1^ Department of Veterinary Preventive MedicineThe Ohio State UniversityColumbusOhio; ^2^ Morris Animal Foundation720 S. Colorado Blvd.Suite 174ADenverColorado; ^3^ Department of Veterinary Preventive Medicine and Public Health Preparedness for Infectious Diseases ProgramThe Ohio State UniversityColumbusOhio

**Keywords:** Antibody test, gold standard, prior distribution, probability intervals, test accuracy, true prevalence

## Abstract

Prevalence of disease in wildlife populations, which is necessary for developing disease models and conducting epidemiologic analyses, is often understudied. Laboratory tests used to screen for diseases in wildlife populations often are validated only for domestic animals. Consequently, the use of these tests for wildlife populations may lead to inaccurate estimates of disease prevalence. We demonstrate the use of Bayesian latent class analysis (LCA) in determining the specificity and sensitivity of a competitive enzyme‐linked immunosorbent assay (cELISA; VMRD ^®^, Inc.) serologic test used to identify exposure to *Neospora caninum* (hereafter *N. caninum*) in three wildlife populations in southeastern Ohio, USA. True prevalence of *N. caninum* exposure in these populations was estimated to range from 0.1% to 3.1% in American bison (*Bison bison*), 51.0% to 53.8% in Père David's deer (*Elaphurus davidianus*), and 40.0% to 45.9% in white‐tailed deer (*Odocoileus virginianus*). The accuracy of the cELISA in American bison and Père David's deer was estimated to be close to the 96% sensitivity and 99% specificity reported by the manufacturer. Sensitivity in white‐tailed deer, however, ranged from 78.9% to 99.9%. Apparent prevalence of *N. caninum* from the test results is not equal to the true prevalence in white‐tailed deer and Père David's deer populations. Even when these species inhabit the same community, the true prevalence in the two deer populations differed from the true prevalence in the American bison population. Variances in prevalence for some species suggest differences in the epidemiology of *N. caninum* for these colocated populations. Bayesian LCA methods could be used as in this example to overcome some of the constraints on validating tests in wildlife species. The ability to accurately evaluate disease status and prevalence in a population improves our understanding of the epidemiology of multihost pathogen systems at the community level.

## Introduction

Surveillance for wildlife diseases is critical to our understanding of the emergence, transmission, persistence, and control of infectious diseases at the interface of humans, domestic animals, and wildlife populations (Jones et al. 2008). For example, describing how the community composition could contribute to the persistence and spread of a multihost pathogen in an ecological system relies directly on the ability to accurately identify the pathogen in the various host populations. Also, accurate measurement of prevalence and incidence in each host population could support ecosystem‐based prevention and control of multihost pathogens, impacting disease dynamics at the community level. However, laboratory tests used to screen diseases in wildlife populations are generally developed for domestic animals and not validated for wildlife species (Gardner et al. 1996). Thus, the application of these tests for wildlife populations may result in inaccurate estimates of epidemiologic parameters. Subsequently, any investigation, analysis or description of a host–pathogen system (e.g., risk factor analysis, transmission modeling) in which estimated parameters are applied might reach biased conclusions (Greiner and Gardner 2000; O'Brien et al. 2004).

Conventionally, the estimation of prevalence of disease in a population is based on validated tests (World Organisation for Animal Health 2013). The *true prevalence* (TP) of disease in a population is the proportion of truly exposed or infected animals, whereas *apparent prevalence* (AP) is the proportion of test‐positive animals (Dohoo et al. 2010). Therefore, the difference between AP and TP of a disease is a function of sensitivity (Se) and specificity (Sp) of the test, where Se is the probability of correctly classifying exposed or infected individuals and Sp is the probability of correctly classifying nonexposed or noninfected individuals. For example, if a test has 50% Sp, half of all nonexposed/ noninfected individuals will test positive (false positives) and will inflate the AP. Conversely, a test with 50% Se would incorrectly classify half of all exposed/ infected individuals (false negatives) and deflate the AP compared with the TP. The relationship among AP, TP, Se, and Sp is TP = [AP + Sp − 1]/[Sp + Se − 1]. Thus, it is important to know the Se and Sp for a test to make correct conclusions about prevalence in a population.

These parameters are often estimated from an experimental population or a reference test (Greiner and Gardner 2000). For wildlife populations, an accurate reference test (referred to as the gold standard) is often unavailable, impractically invasive or prohibitively expensive. Moreover, validation of tests for wildlife populations using traditional field and laboratory methods is limited due to small numbers of individuals and populations, ethical restrictions, economical burden, and specialized management and housing requirements of wildlife species (Gardner et al. 1996). However, Gardner et al. (1996) describe the importance of validating a test for use in wildlife species even when that test has been validated for livestock. Fortunately, statistical methods can be applied to address the issue of uncertain test accuracy in wildlife populations and to estimate prevalence in the absence of a gold standard.

Enoe et al. (2000) discuss the differences and limitations of commonly used statistical methods for maximum likelihood (ML) estimation and Bayesian inference. For instance, they argue that ML estimations rely on the assumption of large sample size; thus, confidence intervals are valid only if that assumption is met. In contrast, Bayesian inference is not restricted by large sample size assumptions (e.g., Normality), but an extra step specifying prior knowledge is required. In the case of wildlife health, large sample sizes are uncommon, but often many small studies or expert opinions can be found, making this an ideal area for use of Bayesian inference.

Bayesian latent class analysis (LCA) combines preliminary estimation of the expected disease prevalence and knowledge of the test performance characteristics with the likelihood of the observed data in order to infer the TP and test Se and Sp of the targeted population. Because a gold standard is not available, the true classification of an individual as infected or not infected is missing; thus, analyses using that latent (or missing) data are called “latent class analysis” (Kaldor and Clayton 1985; Tanner and Wong 1987; Walter and Irwig 1988). As others have shown (Gardner 2004; Branscum et al. 2005; Nielsen et al. 2015; Vilar et al. 2015), this method uses prior knowledge regarding parameter estimates (a *prior distribution*) to circumvent the problem of having two observations (numbers of test‐positive and test‐negative animals) and three unknown parameters to estimate (TP, Se and Sp).

*Neospora caninum* is an excellent case study in which to apply these methods. *N. caninum* is one of the major causes of reproductive problems and abortions in dairy and beef cattle worldwide (Dubey and Schares 2011). This protozoan parasite was first recognized in 1984 in dogs in Norway (Bjerkas et al. 1984). Later, in 1988, it was proposed as a new genus, *Neospora* (Dubey et al. 1988). Currently, the described life cycle of *N. caninum* comprises sylvatic and domestic cycles (Gondim et al. 2004; Almeria 2013). In the United States, coyotes and dogs are believed to be the main definitive hosts and white‐tailed deer and cows the main intermediate hosts (Dubey 2003; Gondim et al. 2004). Numerous investigations have advanced knowledge on host immunology, livestock‐related economics, risk factors, and species exposure to *N. caninum* (Maley et al. 2006; Dubey et al. 2007; Reichel et al. 2013). Although knowledge of exposure of a wide range of species has been described, little is known about the true population prevalence in different species, especially in wildlife; even less is known about *N. caninum*'*s* effects on the population dynamics of these wildlife species and its spread and persistence at the community level. Therefore, estimates of the TP in wildlife populations will advance this area.

Various diagnostic assays have been developed to evaluate *N. caninum* exposure and infection in multiple hosts (Haddad et al. 2005). However, most of these tests are applied in research environments; operational characteristics of the tests might limit their application in surveillance programs (Banoo et al. 2010). For example, time taken to perform tests such as histopathology and immunohistochemistry to assess *N. caninum* in tissue samples (i.e., aborted fetus, placenta, and brain) (Lindsay and Dubey 1989), difficulty of obtaining necessary samples like aborted fetuses from wild ruminants, and technical involvement to identify antibodies in blood by the indirect fluorescent‐antibody assay in various species (Reichel and Pfeiffer 2002) limit their use in wildlife surveillance programs (World Organisation for Animal Health 2014). The commercially available competitive enzyme‐linked immunosorbent assay (cELISA) allows multispecies testing, timely results, easy access, technical simplicity, and an economical way to identify *N. caninum* exposure (Wapenaar et al. 2007). The cELISA serological test has a cutoff value, which maximizes the test Sp and Se for domestic cattle populations (Baszler et al. 2001). However, uncertainty of cELISA accuracy in wildlife populations should be taken into account when estimating prevalence.

Our goal was to infer the TP of *N. caninum* in managed Père David's deer (*Elaphurus davidianus*) and American bison (*Bison bison*) herds and a free‐ranging white‐tailed deer (*Odocoileus virginianus*) population, which inhabit the same community in southeastern Ohio, USA, by applying Bayesian LCA. We have hypothesized that TP will differ by species and from the AP. Also, we used the TP to evaluate the Se and Sp of the commercial cELISA kit (VMRD^®^, Inc., Pullman, WA), which has been used successfully in domestic populations and could identify parasite exposure in these wildlife populations.

## Materials and Methods

### Study area and population

The study area in the Ohio Appalachian bioregion intersects four counties (Muskingum, Morgan, Noble, and Guernsey) and contains the largest conservation center in North America, the International Center for the Preservation of Wild animals (DBA, *the Wilds*). *The Wilds* specializes in captive breeding of rare and endangered species including many endangered ruminant species and encompasses approximately 9250 acres (40.46 km^2^) of reclaimed mine land. The main vegetation is open grassland and forest, (see Dyer (2001) for detailed land structure and vegetation). The area surrounding *the Wilds* is rural, and animal agriculture is the primary or is a supplemental source of income for many families in the area. Free‐ranging wildlife species such as white‐tailed deer (*O. virginianus*) and coyote (*Canis latrans*) are pests, tourist attractions, and a source of food and recreation for the local community. Within *the Wilds*' property, fences limit interactions between livestock and captive wildlife (e.g., American bison and Père David's deer), yet pathogen transmission is plausible between them. White‐tailed deer and coyotes comingle with both captive populations, as both free‐ranging species are capable of crossing many fence lines. This comingling unlocks pathways of pathogen transmission and persistence among Père David's deer, American bison, and white‐tailed deer, our focal species. We have selected this area because it offers a natural laboratory with a complex wildlife–livestock interface that allows us to examine the disease dynamics of multihost pathogen systems, such as *N. caninum*, at the community level.

### Sample collection and testing

We designed a cross‐sectional epidemiological study in which three wildlife species were sampled. We collected tail or jugular vein blood samples (10 mL per individual) during March and April of 2013 for 38 (23 females, 13 males, 2 unrecorded) Père David's deer and 81 (52 females, 26 males, 3 unrecorded) American bison managed at *the Wilds*. The individuals were physically restrained and, once restrained, the procedure lasted about 10 min per animal. Individuals were sampled early in the morning to avoid heat stress and acutely stressed individuals were removed from the study. Thirty samples (27 females and three males) from free‐ranging white‐tailed deer were obtained from the study area during Ohio's hunting season in 2012 and 2013. Deer hunting season in Ohio extends from October to February. We collected 10 ml of blood per deer directly from the heart, soon after death to avoid degradation of antibodies. Animal use protocols were reviewed and approved by the Ohio State University Institutional Animal Care and Use Committee. A white‐tailed deer scientific collection permit was granted by the Division of Wildlife, Ohio Department of Natural Resources. Serum was extracted by centrifugation and stored at −20°C. All species' serum was tested for *N. caninum* antibodies using a commercial cELISA kit (VMRD^®^, Inc.) at the Ohio Department of Agriculture Animal Disease Diagnostic Laboratory, Reynoldsburg, OH. The cELISA was performed according to the manufacturer recommendations. Individual results were reported as percentage inhibition values; a value ≥30% inhibition was considered a positive result and <30% a negative result, as that cutoff is currently used by the manufacturer and has been validated for cattle (Baszler et al. 2001).

### Statistical analysis

Our analysis involved one test across three species to estimate three population parameters (Se, Sp, and prevalence) for each species; thus, there were more parameters to estimate than degrees of freedom in the data (Joseph et al. 1995). Therefore, selection of accurate prior information was essential to minimize the effects of this constraint.

To set prior beliefs, informative and noninformative distributions were used. Informative distributions were based on peer‐reviewed literature estimates of prevalence, Se, and Sp (see Table 1). When 95% confidence intervals were not provided directly by the publication, intervals were approximated accordingly to the “score” method, corrected for continuity (Wilson 1927; Newcombe 1998) using VassarStats: Website for Statistical Computation (Lowry 1998–2015). The parameter space in which prevalence, Se, and Sp fluctuate is from 0 to 1; thus, we used the beta distribution, which is an appropriate family to model uncertainty about parameters within this space (Joseph et al. 1995). For this analysis, transformation of the published estimates to beta parameters (*α*,* β*) was obtained using the “betaExpert” function, available in the package “prevalence” version 0.3.0 built for the statistical program R version 3.1.3 (Devleesschauwer et al. 2014; R Core Team 2015). For noninformative distributions, a beta (*α* = 1, *β* = 1) distribution was used, which gives equal belief to each value within the parameter space. Here, the likelihood of the observed positive and negative test results and the latent data were calculated given prior distributions of prevalence, Se, and Sp as specified in Joseph et al. (1995).

**Table 1:** Published estimates of informative priors

Parameters	Species		
	American bison (*Bison bison*)	Père David's deer (*Elaphurus davidianus*)	White‐tailed deer (*Odocoileus virginianus*)
Prevalence (95% CI)	0.45 (0.02, 2.9)^3^ 13.3 (4.3, 31.6)^3^	25 (11.4, 45.2)^5^	*47.8 (20, 88.2)^2^
Sensitivity (95% CI)	96.4 (92.1, 98.5)^1^ 89 (79, 98)^6^	78.6 (47.7, 96.7)^4^ 80 (29.8, 98.9)^4^	
Specificity (95% CI)	96.8 (92.4, 98.8)^1^ 99 (97, 100)^6^	99 (96.4, 99.7)^4^ 96.6 (93.5, 98.3)^4^	

^1^Baszler et al. (2001); ^2^Dubey et al. (2009); ^3^Dubey and Thulliez (2005); ^4^Pruvot et al. (2014); ^5^Sedlák and Bártová (2006); ^6^Wapenaar et al. (2007).

*The mean prevalence was estimated from all six white‐tailed deer published literature, and the range of prevalence is used versus the 95% confidence interval.

To explore the sensitivity of our conclusions to our choice of prior distributions, eight different estimates (a1–a8) were specified for the American bison herd, five different estimates (b1–b5) were specified for the Père David's deer herd, and five different estimates (c1–c5) were specified for the white‐tailed deer population. Each estimate combines different sets of prior distributions, progressing from noninformative to more informative (see Table 2).

**Table 2:** Models specifications

Model	Species	Prevalence	Sensitivity cELISA	Specificity cELISA
a1	American Bison	Beta (0.29, 63.20)	Beta (1,1)	Beta (1,1)
a2	American Bison	Beta (2.60, 16.92)	Beta (1,1)	Beta (1,1)
a3	American Bison	Beta (1,1)	Beta (104.45, 3.90)	Beta (95.27, 3.15)
a4	American Bison	Beta (1,1)	Beta (38.70, 4.78)	Beta (97.21, 0.98)
a5	American Bison	Beta (0.29, 63.20)	Beta (104.45, 3.90)	Beta (95.27, 3.15)
a6	American Bison	Beta (0.29, 63.20)	Beta (38.70, 4.78)	Beta (97.21, 0.98)
a7	American Bison	Beta (2.60, 16.92)	Beta (104.45, 3.90)	Beta (95.27, 3.15)
a8	American Bison	Beta (2.60, 16.92)	Beta (38.70, 4.78)	Beta (97.21, 0.98)
b1	Père David's deer	Beta (5.65, 16.94)	Beta (1,1)	Beta (1,1)
b2	Père David's deer	Beta (1,1)	Beta (6.85, 1.86)	Beta (103.83, 1.05)
b3	Père David's deer	Beta (1,1)	Beta (2.51, 0.63)	Beta (190.31, 6.70)
b4	Père David's deer	Beta (5.65, 16.94)	Beta (6.85, 1.86)	Beta (103.83, 1.05)
b5	Père David's deer	Beta (5.65, 16.94)	Beta (2.51, 0.63)	Beta (190.31, 6.70)
c1	White‐tailed deer	Beta (3.08, 3.36)	Beta (1,1)	Beta (1,1)
c2	White‐tailed deer	Beta (1,1)	Beta (6.85, 1.86)	Beta (103.83, 1.05)
c3	White‐tailed deer	Beta (1,1)	Beta (2.51, 0.63)	Beta (190.31, 6.70)
c4	White‐tailed deer	Beta (3.08, 3.36)	Beta (6.85, 1.86)	Beta (103.83, 1.05)
c5	White‐tailed deer	Beta (3.08, 3.36)	Beta (2.51, 0.63)	Beta (190.31, 6.70)

The distributions of TP, Se, and Sp were calculated using BayesDiagnosticTest Version 3.9.1 Software Package (Joseph et al. 2015). Concisely, the latent data (numbers of true‐positive and false‐negative individuals) are first estimated, then these estimates are used to obtain the TP, Se, and Sp of the targeted population by applying the Gibbs sampling algorithm. The equations and application of the Gibbs sampling algorithm used in this software are explicitly described in (Joseph et al. 1995). Inferences were based on 100,000 iterations after a discarded burn‐in of 10,000 iterations. The assumptions of the Bayesian estimation procedure we used are convergence and independence. Convergence was assessed by calculating the German Rubin Brooks (BGR) diagnostic, and independence was assessed with autocorrelation plots (all estimates were from procedures that converged and showed independence; diagnostics not shown in this manuscript) using WinBUGS 1.4 software (Lunn et al. 2000; Branscum et al. 2005).

To determine whether the TP differs by species, mode, and 95% probability interval posterior estimates of the three species, pairwise TP comparison was calculated using the most precise prior information for each species.

## Results

A total of 81 American bison, 38 Père David's deer, and 30 white‐tailed deer were sampled. The number of positive individuals was 1/81 for American bison, 26/38 for Père David's deer, and 11/30 for white‐tailed deer when using the 30% inhibition as the cutoff value to discriminate between positive (≥30%) and negative (<30%) samples.

The posterior distributions of prevalence, Se, and Sp by species were summarized by displaying the mode and 95% probability intervals of each of the models (see Tables 3, 4, 5). The models were sensitive for prior distributions; thus, careful selection of prior distributions was needed for inference. To draw inferences on the prevalence, Se, and Sp of our studied populations, models containing informative prior distributions were selected [American Bison (a5–a8); Père David's deer (b4–b5); white‐tailed deer (c4–c5)]. The TP ranges were 0.1 to 3.1%, 51.0 to 53.8%, and 40.0 to 45.9% for the American bison, Père David's deer, and white‐tailed deer, respectively. Se ranges were 90.7% to 97.3%, 95.9 to 99.9%, and 78.9% to 99.9% for the American bison, Père David's deer, and white‐tailed deer, respectively. Sp ranges were 98.2 to 99.9%, 96.7% to 99.9%, and 97.3% to 99.9% for the American bison, Père David's deer, and white‐tailed deer, respectively.

**Table 3:** American Bison herd prior (mean and [95% confidence interval]) and posterior distributions (mode and [95% probability interval])

Model	Prior prevalence	Posterior prevalence	Prior sensitivity	Posterior sensitivity	Prior specificity	Posterior specificity
a1	0.0045 [0.0002, 0.029]	0.001 [2.79E‐08, 0.024]	Non‐informative	0.161 [0.024, 0.974]	Non‐informative	0.988 [0.935, 0.998]
a2	0.133 [0.043, 0.316]	0.108 [0.015, 0.264]	Non‐informative	0.102 [0.006, 0.842]	Non‐informative	0.999 [0.935, 0.999]
a3	Non‐informative	0.011 [5.37E‐04, 0.059]	0.964 [0.921, 0.985]	0.973 [0.921, 0.990]	0.968 [0.924, 0.988]	0.982 [0.953, 0.995]
a4	Non‐informative	0.011 [0.001, 0.070]	0.890 [0.79, 0.98]	0.897 [0.778, 0.963]	0.990 [0.97, 1.00]	0.999 [0.975, 0.999]
a5	0.0045 [0.0002, 0.029]	0.001 [2.09E‐08, 0.017]	0.964 [0.921, 0.985]	0.972 [0.922, 0.990]	0.968 [0.924, 0.988]	0.982 [0.951, 0.994]
a6	0.0045 [0.0002, 0.029]	0.010, 0.011 [4.07E‐08, 0.022]	0.890 [0.79, 0.98]	0.907 [0.783, 0.964]	0.990 [0.97, 1.00]	0.996 [0.971, 0.999]
a7	0.133 [0.043, 0.316]	0.016 [0.006, 0.078]	0.964 [0.921, 0.985]	0.973 [0.920, 0.990]	0.968 [0.924, 0.988]	0.987 [0.954, 0.995]
a8	0.133 [0.043, 0.316]	0.031 [0.008, 0.087]	0.890 [0.79, 0.98]	0.910 [0.773, 0.962]	0.990 [0.97, 1.00]	0.999 [0.977, 0.999]

**Table 4:** Père David's deer herd prior (mean and [95% confidence interval]) and posterior distributions (mode and [95% probability interval])

Model	Prior prevalence	Posterior prevalence	Prior sensitivity	Posterior sensitivity	Prior specificity	Posterior specificity
b1	0.25 [0.114, 0.452]	0.235 [0.103, 0.444]	Non‐informative	0.619, 0.679, 0.877 [0.032, 0.976]	Non‐informative	0.264 [0.035, 0.563]
b2	Non‐informative	0.779 [0.594, 0.986]	0.786 [0.477, 0.967]	0.882 [0.638, 0.974]	0.990 [0.964, 0.997]	0.999 [0.964, 0.999]
b3	Non‐informative	0.715, 0.742 [0.549, 0.979]	0.800 0.298, 0.989]	0.999 [0.642, 0.999]	0.966 [0.935, 0.983]	0.968 [0.936, 0.986]
b4	0.25 [0.114, 0.452]	0.538 [0.403, 0.666]	0.786 [0.477, 0.967]	0.959 [0.787, 0.990]	0.990 [0.964, 0.997]	0.999 [0.959, 0.999]
b5	0.25 [0.114, 0.452]	0.511 [0.383, 0.647	0.800 0.298, 0.989]	0.999 [0.839, 0.999]	0.966 [0.935, 0.983]	0.967, 0.968 [0.932, 0.986]

**Table 5:** White‐tailed deer herd prior (mean and [range]) and posterior distributions (mode and [95% probability interval])

Model	Prior prevalence	Posterior prevalence	Prior sensitivity	Posterior sensitivity	Prior specificity	Posterior specificity
c1	0.478 [0.20, 0.882]	0.378, 0.516 [0.152, 0.817]	Non‐informative	0.254 [0.025, 0.929]	Non‐informative	0.664, 0.744 [0.086, 0.973]
c2	Non‐informative	0.397 [0.257, 0.859]	0.786 [0.477, 0.967]	0.902 [0.4668, 0.9691	0.990 [0.964, 0.997]	0.999 [0.964, 0.999]
c3	Non‐informative	0.423 [0.222, 0.916]	0.800 [0.298, 0.989]	0.999 [0.379, 0.9985]	0.966 [0.935, 0.983]	0.969 [0.936, 0.986]
c4	0.478 [0.20, 0.882]	0.459 [0.273, 0.730]	0.786 [0.477, 0.967]	0.789, 0.859 [0.512, 0.969]	0.990 [0.964, 0.997]	0.999 [0.964, 0.999]
c5	0.478 [0.20, 0.882]	0.401 [0.243, 0.747]	0.800 [0.298, 0.989]	0.999 [0.442, 0.999]	0.966 [0.935, 0.983]	0.973 [0.936, 0.986]

Models a7, b4, and c4 were selected to compare AP and TP and to test whether TP differed by species. Selection of these three models was grounded on informed prior distributions and two main factors (1) prior data were from species belonging to the same family and/or (2) population management was similar in the herd used in the prior estimate. AP was greater than TP in Père David's deer and less than TP in white‐tailed deer, while only a slight difference was seen between AP and TP in American bison (see Table 6).

**Table 6:** Apparent prevalence versus true prevalence

Species	Apparent prevalence [95% CI]	True prevalence* [95% PI]
American Bison	1.23% [0.06, 7.6]	1.6% [0.6, 7.8]
Père David's deer	68.4% [51, 82]	53.8% [40.3, 66.6]
White‐tailed deer	36.7% [20.5, 56]	45.9% [27.3, 73]

*The mode of models a7, b4, and c4 were selected to represent true prevalence.

**Figure 1:**
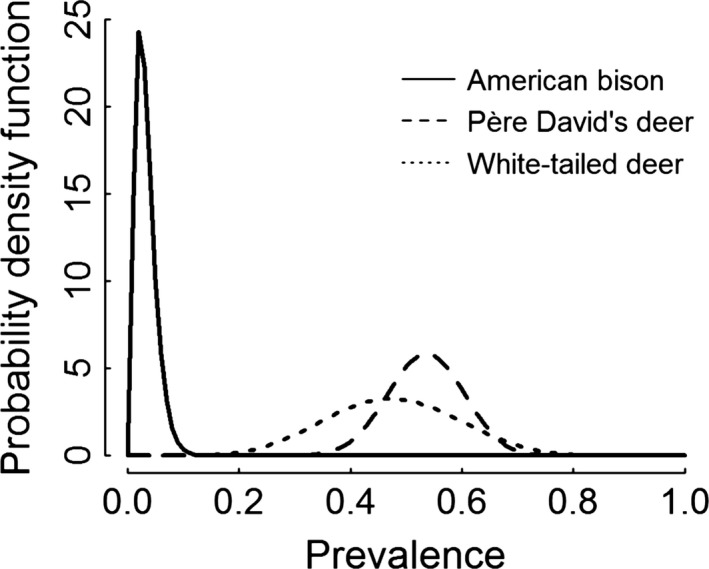
Compared true prevalence by species. Models a7, b4, and c4 were selected to represent true prevalence. The mode of true prevalence was 1.6% for American bison, 53.8% for Père David's deer, and 45.9% for white‐tailed deer.

We found that TP differed between American bison and the two deer populations. However, the TP was similar between Père David's deer and white‐tailed deer (Fig. 1, Table 7).

**Table 7:** The three species pairwise true prevalence comparison

	Difference of the pairwise species comparison Mode [95%PI]
Père David's deer–American Bison	0.49 [0.36 to 0.63]
White‐tailed deer–American Bison	0.46 [0.23 to 0.69]
White‐tailed deer–Père David's deer	−0.11 [−0.30 to 0.22]

Models a7, b4, and c4 were selected to determine the difference of the TP by species.

## Discussion

In our study, we estimated TP, Se, and Sp of *N. caninum* exposure in three wildlife species in southeastern Ohio, USA. The accuracy of the commercially available cELISA kit (VMRD^®^, Inc.) in these species, using the 30% cutoff value, was close to the 96% Se and 99% Sp that the manufacturer specifies. High Sp was observed for all species tested, which makes this test good for diagnostic purposes. However, low Se was observed for white‐tailed deer; thus, surveillance programs need to take into account that some infected animals may not test positive using the 30% inhibition cutoff criterion, or more work should be performed in this species to establish the true Se of the test compared with a gold standard.

Next, we compared AP versus TP. Apparent prevalence overestimated TP in Père David's deer and underestimated TP in white‐tailed deer, while only a slight difference was seen between AP and TP in American bison. Despite the availability of methods to measure disease prevalence when diagnostic uncertainties are unknown, AP continues to be reported (Guatteo et al. 2011; Lewis and Torgerson 2012). Apparent prevalence measured over time might provide the trajectory of a disease in a population. However, AP might hinder control or eradication programs. For example, a study on bovine tuberculosis in harvested white‐tailed deer in Michigan showed that AP used as a metric in the surveillance program to assess progress toward disease eradication underestimated TP. Hence, false‐negative individuals were hindering a control program and a risk to hunters' health (O'Brien et al. 2004). Because the goal of surveillance programs is to detect infected individuals, a more sensitive test would be preferred, especially when prevalence is low, or the test results must be adjusted. Additionally, if AP is skewed differently for different species as appears to be the case here, conclusions of metapopulation transmission models may be inaccurate if they use the skewed data across populations.

Finally, although these species inhabit the same community, we found that TP differed between American bison and the two deer populations, but TP was similar between Père David's deer and white‐tailed deer. Assuming that ruminant's exposure to *N. caninum* from environmental contamination is homogenously distributed across the community, the variations seen among species might relate to difference in management (e.g., number of individuals transferred out of and into the population), differences in behavior (e.g., matriarchal structure of a population), and differences in immunity (e.g., host susceptibility or efficiency of vertical transmission). In 2003, a study estimated an overall cattle sero‐positivity of 4.7% in Ohio and 9.2% in southeastern Ohio (Hinrichs 2003); thus, Neospora‐endemic regions such as ours might further benefit from the correct classification of the health status of individuals. Correct classification might add better understanding on the role of types of host (e.g., maintenance host, spillover host), as well as quantifying the between‐ and within‐species transmission on the persistence of a pathogen in a community (Fenton and Pedersen 2005).

Although Bayesian LCA overcomes many constraints of test validation in wildlife species, careful selection of prior distributions is needed because of unidentifiability (more parameters to estimate than degrees of freedom), which may lead to many distinct values of the parameters. For instance, we found that estimation using noninformative combinations of prior distributions (those giving equal belief to a prevalence of zero and a prevalence of one) produced very different estimates of prevalence, Se, and Sp, than did our informed prior distributions (see Tables 3, 4, 5). Thus, we used estimates of the population parameters where prior distributions were informative, that is, they restricted the parameter space to values that seem reasonable based on literature. When we used different informed prior distributions, parameter estimates did not vary widely.

In our study, previous information on prevalence was available for the three species studied. However, prior information for Se and Sp was obtained from experimental data studying cows, which are a reasonable surrogate for American bison as both belong to the *Bovidae* family, and a statistical validation studying elk, which are a reasonable surrogate for the two deer populations, as all belong to the *Cervidae* family.

In terms of the quantification of *N. caninum* exposure in wildlife populations, a variety of serological tests have been implemented, nevertheless this diversity disfavors the comparison of the prevalence estimates, due to the various methods and cutoff values used among researchers (Wapenaar et al. 2007; Dubey et al. 2009). Additionally, uncertainty around those estimates is often unidentified.

Modeling the uncertainty about the values for test accuracy and prevalence with probability provides a rational view of dealing with incomplete knowledge or knowledge that may peripherally influence beliefs about test characteristics (Enoe et al. 2000). For example, we accounted for uncertainty on cross‐reaction by allowing a range of probabilities in prior distributions of Sp. Cross‐reaction with antibodies produced by antigenically related parasites such as *Toxoplasma gondii*,* Sarcocystis cruzi*,* Sarcocystis hominis*, and *Sarcocystis hirsute* could reduce test Sp; however, the use of the 65‐kDa *N. caninum* tachyzoite antigen and a monoclonal antibody (MAb 4A 4‐2) in the competitive ELISA (VMRD^®^, Inc.) to detect antibodies in sera instead of whole tachyzoite antigens used in indirect ELISA and IFA tests has been suggested to alleviate this problem (Baszler et al. 1996). Nevertheless, a serological cross‐reaction with *Hammondia heydorni* has not been evaluated (Gondim 2006).

To our knowledge, the cELISA (VMRD^®^, Inc.) for *N. caninum* has been experimentally validated for cattle, statistically validated for elk, and partially validated for dogs through the work of Baszler et al. (2001), Pruvot et al. (2014), King et al. (2012), and Capelli et al. (2006). Our results expand validation of the use of this test for white‐tailed deer, American bison, and Père David's deer. The geographical distribution and abundance of white‐tailed deer in America, in addition to hunting practices, make this potential intermediate host a valuable target species for understanding and controlling *N. caninum* at the wildlife–livestock interface (Gondim 2006). With regard to American bison and Père David's deer, conservation efforts might be hindered by the persistence of this pathogen in their populations. Thus, better estimates of exposure might better explain the role of these species in the transmission cycle of *N. caninum* and the effects of the pathogen on reproduction, not only at the population level but also at the community level.

Variation of *N. caninum* prevalence among cohabiting species demonstrates likely differences in disease dynamics for the different species; thus providing evidence for the importance of community‐based approaches to understanding transmission and persistence of multihost pathogens.

## Data Accessibility

Data are available and clearly written in the article.

## Conflict of Interest

None declared.
